# Exploratory Investigation of Predictive Factors for Reducing Adverse Effects in CapeOX Therapy for Colorectal Cancer Patients

**DOI:** 10.7759/cureus.95702

**Published:** 2025-10-29

**Authors:** Makoto Kishimoto, Masayoshi Koinuma, Jin Tokunaga

**Affiliations:** 1 First Department of Clinical Pharmacy, Faculty of Pharmaceutical Sciences, Kyushu University of Medical Science, Nobeoka, JPN; 2 Department of Pharmacy, Kirishima Medical Center, Kirishima, JPN; 3 Faculty of Pharmaceutical Sciences, Teikyo Heisei University, Tokyo, JPN

**Keywords:** adverse effect, capeox therapy, colorectal cancer, cut-off value, inflammatory marker, predictor

## Abstract

Background: Oxaliplatin infusion plus oral capecitabine (CapeOX) is the standard chemotherapy regimen for colorectal cancer. It is commonly employed as adjuvant therapy and as first-line treatment for advanced disease. However, adverse effects, such as peripheral neuropathy, hand-foot syndrome, and gastrointestinal symptoms, may lead to dose reduction or discontinuation. Identifying reliable predictors of these adverse effects would support prophylactic strategies and enable safer, more personalized treatment.

Methods: We retrospectively analyzed 108 colorectal cancer patients who received CapeOX between September 2020 and October 2023. Adverse events after the first cycle were evaluated using Common Terminology Criteria for Adverse Events (CTCAE) v5.0 and categorized into Grade 0 and Grade ≥ 1 groups. Associations between pre-treatment blood test values (including aspartate aminotransferase (AST), alanine aminotransferase (ALT), red blood cells, monocytes, and basophils) and inflammatory or nutritional indices (including prognostic nutrition index (PNI), systemic inflammatory response index (SIRI), neutrophil to lymphocyte ratio (NLR), platelet to lymphocyte ratio (PLR)) were examined for adverse events. Variables demonstrating significant associations in univariate analysis were included in a multivariate logistic regression model. Receiver operating characteristic analysis was performed to determine optimal cut-off values of predictive markers.

Results: AST ≤ 22.0 IU/L was identified as a significant predictor of peripheral neuropathy. Nausea was significantly associated with a basophil count ≥ 0.28 × 10²/μL and occasional alcohol intake; anorexia with a basophil count ≥ 0.29 × 10²/μL and PLR ≤ 152.69; and diarrhea with a SIRI ≤ 0.37. These markers enabled effective risk stratification of adverse drug reactions in patients undergoing CapeOX.

Conclusions: Certain adverse effects of CapeOX may be predicted using routine pre-treatment laboratory data. These findings could facilitate early identification of high-risk patients and enable personalized preventive interventions.

## Introduction

In Japan in 2020, colorectal cancer ranked second in incidence among both males and females, and first overall in total number of cancer cases. In terms of the number of deaths, it ranks second in men and first in women, and second in the total number of deaths [1]. The standard chemotherapy for colorectal cancer is oxaliplatin infusion plus oral capecitabine (CapeOX) [2]. CapeOX is also the first-line systemic therapy for patients with unresectable advanced disease or distant metastases.

Chemotherapy induces a variety of adverse effects, including nausea, vomiting, diarrhea, neurotoxicity, and neutropenia. Severe adverse effects may lead to prolongation or discontinuation of treatment. Therefore, accurate prediction of adverse effect onset would enable timely intervention while symptoms are mild, helping to prevent treatment delays or discontinuation. We conducted a survey of high-risk adverse effects associated with CapeOX therapy, aiming to identify predictors of these adverse effects from routinely collected clinical data, including blood test parameters.

## Materials and methods

Study design and patients

This retrospective study analyzed data from colorectal cancer patients who received CapeOX therapy between September 1, 2020, and October 31, 2023, at Kirishima City Medical Association Medical Center. Exclusion criteria were: (1) patients who declined to participate in the study, and (2) patients who discontinued treatment at their own discretion. Patients were excluded if variables necessary for analysis (such as blood test values or adverse event information) were missing. Accordingly, 59 men and 49 women were included in the final analysis.

Ethical approval for this study was obtained from the Ethical Review Committee for Clinical Research of Kirishima City Medical Center (Receipt No. 202301) and the Ethical Review Committee of Kyushu University of Medical Science (Approval No. 23-015). Due to the retrospective design, the need for written informed consent was waived, and an opt-out process was implemented through public disclosure on the hospital website.

Data collection

Relevant data were collected retrospectively from the electronic medical record system, including: (1) patient information, including age, sex, body mass index, body surface area, general condition (Eastern Cooperative Oncology Group Performance Status), smoking history, alcohol consumption history, primary site, stage, oxaliplatin dose, and capecitabine dose; (2) blood laboratory parameters, including red blood cells, white blood cells, basophils, eosinophils, neutrophils, lymphocytes, monocytes, platelet count, serum albumin level (Alb), C-reactive protein, aspartate aminotransferase (AST), alanine aminotransferase (ALT), hemoglobin level (Hb), estimated glomerular filtration rate, serum creatinine, and creatinine clearance; (3) adverse effects of chemotherapy, including peripheral neuropathy, hand-foot syndrome, nausea, anorexia, diarrhea, leukopenia, neutropenia, hemoglobin depletion, thrombocytopenia, eye disorder, taste disorder, general fatigue, digestive symptoms (heartburn), and liver dysfunction; and (4) nutrition and inflammatory markers, including prognostic nutrition index (PNI), systemic inflammatory response index (SIRI), inflammatory indices (neutrophil to lymphocyte ratio [NLR], platelet to lymphocyte ratio [PLR], lymphocyte to monocyte ratio [LMR], and systemic immune inflammatory index [SII]).

PNI was calculated as 10 × Alb (g/dL) + 0.005 × total lymphocyte count (/mm^3^). SIRI was calculated as neutrophils (10^2^/μL) × monocytes (10^2^/μL)/lymphocytes (10^2^/μL). NLR was calculated as neutrophils (10^2^/μL)/lymphocytes (10^2^/μL). PLR was calculated as platelets (10^4^/μL)/lymphocytes (10^2^/μL). LMR was calculated as lymphocytes (10^2^/μL)/monocytes (10^2^/μL). SII was calculated as platelets (10^4^/μL) × (neutrophils [10^2^/μL])/lymphocyte count [10^2^/μL]).

Study endpoint and assessment of adverse drug reactions

All patients were chemotherapy-naïve and received one cycle of CapeOX therapy. CapeOX therapy consisted of intravenous oxaliplatin (130 mg/m^2^) on day one and oral capecitabine (1,000 mg/m^2^ twice daily) on days one to 14, for a 21-day cycle.

The primary endpoint of the study was the occurrence of grade 1 or higher adverse reactions at the end of one cycle. The grade of adverse reactions was assessed based on the Common Terminology Criteria for Adverse Events (CTCAE) v5.0 Japanese translation.

Statistical analyses

Each adverse effect at the end of one cycle was divided into Grade 0 or Grade ≥ 1 groups. We searched for predictors of the relevant adverse effects by comparing patient information, blood laboratory values, PNI, SIRI, and inflammatory indices (NLR, PLR, LMR, and SII).

Multivariable logistic regression analysis was conducted on factors that showed significant differences in univariate analysis. If only one factor was significant, a multivariate analysis was performed including that factor and the one with the second-lowest p-value. Drinking history (none, occasional, weekly, daily) was treated as a nominal variable and converted into dummy variables, with 'no drinking' as the reference category.

Receiver operating characteristic (ROC) curves were used to set cut-off values for factors that showed significant differences. Based on the cut-off values, the patient population was divided into two groups for analysis. All continuous variables were expressed as mean ± standard deviation. Categorical variables were presented as integers and percentages. The Wilcoxon two-sample test, Pearson's chi-square test, and Fisher's exact test were used for analysis. All p-values were two-tailed with a dominance level of 0.05. All statistical tests were performed using JMP® version 17 (SAS Institute Inc., Cary, NC, USA).

## Results

Patient characteristics

A total of 108 colorectal cancer patients (59 men, 49 women) with a mean age of 66.8 years (61.0-74.3) were included. The following adverse effects of Grade 1 or higher were observed during CapeOX therapy (oxaliplatin + capecitabine): peripheral neuropathy (48 patients, cumulative oxaliplatin dose 89.8-130.1 mg/m², capecitabine 1,208-2,186 mg/m² per cycle); hand-foot syndrome (12 patients, oxaliplatin 99.8-128.7 mg/m², capecitabine 1,548-2,000 mg/m²); nausea (nine patients, oxaliplatin 103.2-128.7 mg/m², capecitabine 1,518-2,128 mg/m²); anorexia (12 patients, oxaliplatin 100.8-127.9 mg/m², capecitabine 1,550-2,110 mg/m²); diarrhea (four patients, oxaliplatin 97.5-127.0 mg/m², capecitabine 1,376-1,904 mg/m²); leukopenia (11 patients); neutropenia (15 patients); Hb decreased (37 patients); thrombocytopenia (26 patients); general fatigue (18 patients); taste disorder (five patients); eye disorder (two patients); digestive symptoms such as heartburn (two patients); and liver dysfunction (one patient). Among them, the adverse reactions that showed significant differences in patient information, blood laboratory values, PNI, SIRI, or inflammatory indices between the Grade 0 and Grade ≥ 1 groups at the beginning of one cycle were: peripheral neuropathy, hand-foot syndrome, nausea, anorexia, diarrhea, leukopenia, neutropenia, Hb decreased, and thrombocytopenia. We examined predictive factors for peripheral neuropathy, hand-foot syndrome, nausea, anorexia, and diarrhea (Table 1). For adverse effects with very low incidence (e.g., eye disorder, heartburn, liver dysfunction), the small number of cases limits statistical analysis and should be interpreted with caution. The total number of patients with each adverse event category (Grade 0 and Grade ≥1) corresponds to the full cohort of 108 patients, with no missing data for these variables. Among patients with a history of smoking, 78.5% were male and 21.5% were female. Similarly, for drinking habits, everyday drinkers were predominantly male (88.6%), while occasional drinkers included both sexes equally. Weekly drinkers also showed a male predominance, with 83.3% being male.

**Table 1:** Baseline patient information by adverse effect observed at the end of one treatment cycle Values marked with an asterisk (*) are statistically significant (p < 0.05). Statistical tests used: a) Wilcoxon two-sample test; b) Pearson's chi-square test.

Variable		Peripheral neuropathy			Hand-foot syndrome			Nausea			Anorexia			Diarrhea			
		Grade 0	Grade ≥ 1	p-value	Grade 0	Grade ≥ 1	p-value	Grade 0	Grade ≥ 1	p-value	Grade 0	Grade ≥ 1	p-value	Grade 0	Grade ≥ 1	p-value	
		n = 60	n = 48		n = 96	n = 12		n = 99	n = 9		n = 96	n = 12		n = 104	n = 4		
Age (years)		66.81±9.73	66.87±10.79	0.7266	66.66±10.21	68.25±10.56	0.463	67.53±9.52	59.22±14.60	0.1116	66.97±10.42	65.75±8.66	0.4308	66.73±10.33	69.75±6.39	0.6661	a)
Sex	Male	34	25	0.6345	53	6	0.7326	54	5	0.9535	52	7	0.4846	56	3	0.4043	b)
	Female	26	23		43	6		45	4		44	5		48	1		
BMI (kg/m²)		22.12±3.67	22.38±3.98	0.9556	22.20±3.68	22.49±4.89	0.8873	22.12±3.71	23.52±4.88	0.7055	22.19±3.73	22.62±4.58	0.895	22.24±3.87	22.10±2.27	0.7759	a)
Body surface area (m²)		1.56±0.18	1.58±0.20	0.7572	1.57±0.19	1.56±0.22	0.6707	1.57±0.19	1.61±0.22	0.6248	1.57±0.19	1.58±0.15	0.9649	1.57±0.19	1.60±0.13	0.6902	a)
General condition（PS）	0	51	44	0.0644	86	11	0.8175	90	7	0.2124	86	11	0.822	93	4	0.4925	b)
	1	9	4		10	1		9	2		10	1		11	0		
Smoking history	0	24	19	0.9901	40	3	0.2893	41	2	0.0784	40	3	0.1545	42	1	0.3867	b)
	1-199	5	5		8	2		7	3		7	3		10	0		
	200-399	4	4		7	1		7	1		7	1		8	0		
	400-699	8	6		14	6		14	0		14	0		14	0		
	700-	19	14		27	6		30	3		28	5		30	3		
Drinking history	None	18	2	0.9276	31	3	0.5549	33	1	0.0240*	33	1	0.3297	34	0	0.2222	b)
	Occasional	13	8		19	2		16	5		18	3		20	1		
	Weekly	10	8		17	1		16	2		15	3		18	0		
	Everyday	19	16		29	6		34	1		30	5		32	3		
Cancer site	Upper colon	11	7	0.1024	11	0	0.6818	10	1	0.9928	11	0	0.4608	11	0	0.6974	b)
	Lower colon	5	6		5	1		6	0		5	1		6	0		
	Sigmoid colon	23	16		21	3		22	2		19	5		22	2		
	Transverse colon	17	7		15	4		17	2		17	2		19	0		
	Jejunum	1	1		1	0		1	0		1	0		1	0		
	Rectum	28	22		33	3		33	3		34	2		35	1		
	Cecum	8	6		10	1		10	1		9	2		10	1		
Stage	Ⅱa	15	9	0.4817	21	3	0.7087	24	0	0.1573	22	2	0.9014	23	1	0.9987	b)
	Ⅱb	5	1		6	0		6	0		5	1		6	0		
	Ⅲa	7	7		12	2		14	0		14	0		14	0		
	Ⅲb	22	21		38	5		37	6		37	6		40	3		
	Ⅲc	5	6		10	1		9	2		8	3		11	0		
	Ⅳ	6	4		9	1		9	1		10	0		10	0		
Dose(%)	Oxaliplatin	88.94±7.91	89.20±7.98	0.8359	89.41±7.97	86.21±7.06	0.1466	89.17±8.04	87.78±6.54	0.5336	89.03±7.95	89.22±7.89	0.9805	89.00±7.85	90.60±10.58	0.597	a)
	Capecitabine	93.29±10.15	92.89±9.75	0.9359	93.71±9.85	88.35±10.14	0.1412	93.30±9.95	91.10±10.64	0.6091	92.88±10.07	94.98±9.36	0.4605	93.44±9.83	84.64±11.62	0.1091	a)

Peripheral neuropathy

AST (IU/L) at the start of one cycle was significantly lower in the Grade ≥ 1 group (17.70 ± 4.55, n = 60) than in the Grade 0 group (23.70 ± 14.02, n = 48) (mean difference: 6.00 IU/L, 95% CI: 1.86-10.14, p = 0.0128) (Table 2). ALT (IU/L) at the beginning of one cycle was significantly lower in the Grade ≥ 1 group (16.95 ± 6.87, n = 60) than in the Grade 0 group (22.70 ± 13.89, n = 48) (mean difference: 5.75 IU/L, 95% CI: 1.46-10.04, p = 0.0378) (Table 2). Multivariable logistic regression analysis identified AST (odds ratio [OR] = 0.9272, 95% CI: 0.8481-0.9961, p = 0.0371) as an independent factor associated with the development of peripheral neuropathy (Table 3). On the other hand, no significant association was found with ALT (p > 0.05) (Table 3).

**Table 2:** Comparison of blood test values, nutritional and inflammatory indices before treatment by adverse effect after one cycle Values marked with an asterisk (*) are statistically significant (p < 0.05). Statistical tests used: Wilcoxon two-sample test. CRP: C-reactive protein; AST: Aspartate Aminotransferase; ALT: Alanine aminotransferase; Hb: Hemoglobin; eGFR: estimated Glomerular Filtration Rate; Cr: Creatinine; Ccr: Creatinine clearance; PNI: prognostic nutrition index; SIRI: systemic inflammatory response index; NLR; neutrophil to lymphocyte ratio; PLR: platelet to lymphocyte ratio; LMR: lymphocyte to monocyte ratio; SII: systemic immune inflammatory index.

Variable	Reference range (normal value)	Peripheral neuropathy			Hand-foot syndrome			Nausea			Anorexia			Diarrhea		
		Grade 0	Grade ≥ 1	p-value	Grade 0	Grade ≥ 1	p-value	Grade 0	Grade ≥ 1	p-value	Grade 0	Grade ≥ 1	p-value	Grade 0	Grade ≥ 1	p-value
		n = 60	n = 48		n = 96	n = 12		n = 99	n = 9		n = 96	n = 12		n = 104	n = 4	
Red blood cell (10^4^/μL)	Male 420–570 ／ Female 380–500	423.5±51.97	411.52±78.95	0.9901	413.28±57.19	353.91±107.37	0.0472*	421.14±56.23	385.55±130.31	0.6169	421.44±55.70	392.00±117.62	0.66	417.91±65.68	425.00±62.10	0.9416
																	White blood cell (10^2^/μL)	35–90	56.38±17.82	63.58±56.36	0.8504	49.41±16.39	82.00±112.38	0.4368	56.38±17.10	94.77±126.79	0.9557	56.46±16.81	84.50±110.45	0.7102	59.93±40.31	50.50±23.95	0.459
Basophil (10^2^/μL)	0-2	0.27±0.20	0.35±0.31	0.0576	0.012±0.110	0.00±0.00	0.6302	0.28±0.22	0.56±0.49	0.0297*	0.28±0.22	0.52±0.40	0.0018*	0.30±0.26	0.49±0.26	0.1431
																	Eosinophil (10^2^/μL)	0-6	1.93±1.30	2.39±2.84	0.6878	1.65±1.75	5.34±6.07	0.0698	1.98±1.32	3.82±5.99	0.8939	1.94±1.34	3.69±5.06	0.0683	2.15±2.16	1.80±0.95	0.8135
Neutrophil (10^2^/μL)	40-75	34.35±14.95	39.43±38.49	0.6362	27.24±11.82	45.13±64.55	0.5807	34.21±13.73	62.95±85.30	0.3225	34.76±13.75	51.36±75.44	0.5674	36.94±28.32	28.02±13.79	0.3054																	
																	Lymphocyte (10^2^/μL)	20-50	16.11±6.07	17.43±13.22	0.8967	16.02±5.55	25.31±33.25	0.625	16.20±5.77	22.17±29.33	0.2343	15.82±5.60	23.77±24.82	0.4302	16.67±9.96	17.44±8.46	0.6664
Monocyte (10^2^/μL)	2-10	3.69±1.43	3.96±2.83	0.9926	4.23±1.88	7.60±8.50	0.0477*	3.68±1.39	5.24±6.03	0.8632	3.64±1.34	5.14±5.28	0.8069	3.85±2.18	2.73±1.27	0.1164																	
																	Platelet (10^4^/μL)	15-35	25.63±7.72	24.56±7.86	0.4433	20.19±6.21	20.35±7.01	0.788	25.00±7.64	26.85±9.36	0.4977	25.58±7.94	21.72±5.29	0.1521	25.31±7.77	21.15±7.41	0.6026
Serum Albumin (g/dL)	3.8-5.0	4.03±0.50	4.07±0.39	0.8184	4.07±0.37	3.85±0.23	0.0219*	4.05±0.47	4.04±0.26	0.7337	4.03±0.46	4.20±0.35	0.2372	4.04±0.45	4.17±0.56	0.7689																	
																	CRP (mg/dL)	<0.3	0.38±1.40	0.31±0.50	0.9529	0.34±1.26	0.51±0.92	0.8596	0.35±1.13	0.28±0.45	0.3311	0.36±1.15	0.27±0.40	0.5135	0.35±1.11	0.19±0.16	0.5344
AST (IU/L)	13-33	23.70±14.02	17.70±4.55	0.0128*	30.66±53.64	19.33±5.19	0.0469*	21.06±11.64	20.77±5.51	0.4004	21.27±11.82	19.16±4.28	0.9142	21.26±11.39	15.00±2.16	0.077																	
																	ALT (IU/L)	8-42	22.70±13.89	16.95±6.87	0.0378*	23.29±14.30	15.08±9.41	0.0090*	19.59±11.34	26.22±13.78	0.0896	19.83±11.51	22.66±12.78	0.3731	20.26±11.73	17.00±9.12	0.5413
Hb (g/dL)	Male 13.0–17.0 ／ Female 11.5–15.0	12.89±3.99	12.35±1.73	0.6673	12.67±3.36	11.9±1.24	0.3256	12.68±3.31	12.30±1.33	0.8067	12.63±3.34	12.82±1.62	0.4369	12.65±3.24	12.82±1.52	0.6486																	
																	eGFR (mL/min/1.73m^2^)	≥60	76.66±19.63	78.43±20.77	0.7571	78.45±19.61	79.75±13.95	0.8872	77.43±19.80	77.66±24.19	0.5262	77.67±20.97	75.66±10.77	0.7322	77.49±20.44	76.50±4.12	0.8643
Cr (mg/dL)	Male 0.65–1.07 ／ Female 0.46–0.79	0.71±0.17	0.70±0.16	0.963	0.70±0.16	0.66±0.12	0.8106	0.70±0.17	0.74±0.15	0.5335	0.70±0.17	0.72±0.16	0.6042	0.70±0.17	0.72±0.06	0.558																	
																	Ccr (mL/min)	80-130	160.65±42.03	164.19±44.55	0.9286	162.77±40.03	166.95±49.19	0.7656	161.90±41.26	165.84±62.17	0.8895	162.66±43.53	158.74±40.08	0.7881	162.50±43.71	155.03±17.36	0.7265
PNI	≥45(Good)	48.39±6.04	49.42±7.53	0.8311	48.78±4.72	51.23±16.76	0.1563	48.60±5.65	51.53±14.38	0.7896	48.22±5.44	53.88±12.42	0.1189	48.79±6.68	50.47±8.95	0.8644																	
SIRI	1.0-1.5(clinical cut-off)	0.91±0.67	0.95±0.93	0.7038	0.82±0.69	1.37±1.68	0.1696	0.87±0.63	1.47±1.75	0.117	0.90±0.64	1.11±1.59	0.6146	0.94±0.80	0.44±0.21	0.0412*
NLR	1.0-3.0	2.37±1.23	2.32±0.84	0.559	1.84±0.94	1.73±0.51	0.7731	2.31±1.10	2.80±0.53	0.0310*	2.40±1.11	1.93±0.51	0.2199	2.38±1.08	1.65±0.31	0.141
PLR	100-200	178.51±77.58	164.88±67.11	0.5182	138.22±60.83	127.36±69.16	0.8297	169.92±69.91	200.27±103.13	0.1934	178.01±73.45	127.98±54.31	0.0242*	174.14±73.84	128.48±26.05	0.1796
LMR	3.0-5.0	4.85±2.29	4.65±1.57	0.9852	4.30±2.02	3.29±0.65	0.0698	4.82±2.05	4.17±1.21	0.4235	4.73±2.00	4.99±2.02	0.685	4.69±0.19	6.57±2.71	0.0954
SII	≤500(Increased by inflammation and tumors)	626.98±433.74	567.47±291.35	0.9803	380.00±262.47	358.17±160.30	0.5412	584.94±373.94	771.97±385.98	0.0559	624.04±390.95	412.41±130.09	0.0612	610.70±379.88	336.09±99.09	0.0861

**Table 3:** Multivariable Logistic Regression Analysis of Significant Factors Values marked with an asterisk (*) are statistically significant (p < 0.05). Statistical tests used: Multivariable Logistic Regression Analysis. Note: Drinking status was treated as a categorical variable, with "non-drinker" as the reference category. Odds ratios (OR) were calculated relative to non-drinkers. AST: Aspartate Aminotransferase; ALT: Alanine aminotransferase; NLR; neutrophil to lymphocyte ratio; PLR: platelet to lymphocyte ratio; SIRI: systemic inflammatory response index.

		Odds ratio	95％CI	p-value
(A) Peripheral neuropathy				
AST (IU/L)		0.9272	0.8481-0.9961	0.0371*
					ALT (IU/L)		0.9781	0.9237-1.0326	0.4271
(B) Hand-foot syndrome									
Red blood cell (10^4^/μL)		0.9911	0.9781-1.0027	0.1369
					Monocyte (10^2^/μL)		1.2109	0.8640-1.8488	0.3077
Serum Albumin (g/dL)		1.2773	0.2683-7.3249	0.7654					
					AST (IU/L)		0.9743	0.8500-1.0583	0.6084
ALT (IU/L)		0.9916	0.9004-1.0825	0.8537					
					(C) Nausea				
Drinking history	Occasional	10.3125	1.5016-206.3543	0.0157*
	Weekly	4.125	0.3690-92.6518	0.2438
	Everyday	0.9706	0.0374-25.2063	0.9834
Basophil (10^2^/μL)		12.0921	1.7492-114.2699	0.0138*
					NLR		1.5417	0.8039-2.8308	0.1649
(D) Anorexia				
Basophil (10^2^/μL)		7.3613	1.0601-73.0202	0.0434*
					PLR		0.3282	0.0827-0.9694	0.0430*
(E) Diarrhea				
SIRI		0.9941	0.9848-0.9999	0.0420*
AST (IU/L)		0.8151	0.5706-1.0153	0.0752

The discriminative ability of the model was assessed using ROC curve analysis for AST, yielding an area under the curve (AUC) of 0.64, which indicates limited predictive performance (Table 4). The cut-off value was 22.0 (95%CI: 15.0-23.0) (Table 4); thus, Grade ≥ 1 peripheral neuropathy occurred significantly at ASTs below 22.0 IU/L (p = 0.000145).

**Table 4:** Significant predictors and cutoff values by adverse effect, and comparisons before and after cutoff Values marked with an asterisk (*) are statistically significant (p < 0.05). Statistical tests used: Fisher's exact test. AST: Aspartate Aminotransferase; PLR: platelet to lymphocyte ratio; SIRI: systemic inflammatory response index; AUC: area under the curve

	Cut-off value	AUC	95％CI		Grade0	Grade≥1	p-value
(A) Peripheral neuropathy							
AST (IU/L)	22	0.63976	15.0-23.0	≤22.0	32 (43.2%)	42 (56.8%)	0.000145*
				>22.0	28 (82.3%)	6 (17.7%)	
(B) Nausea							
Basophil (10^2^/μL)	0.28	0.71998	0.3-0.7	<0.28	55 (98.2%)	1 (1.8%)	0.0137*
				≥0.28	44 (84.6%)	8 (15.4%)	
(C) Anorexia							
Basophil (10^2^/μL)	0.29	0.77821	0.3-0.7	<0.29	60 (98.4%)	1 (1.6%)	0.0004*
				≥0.29	36 (76.6%)	11 (23.4%)	
PLR	152.69	0.70052	1.13-2.11	≤152.69	40 (81.6%)	9 (18.4%)	0.0137*
				>152.69	56 (94.9%)	3 (5.1%)	
(D) Diarrhea							
SIRI	0.372	0.80288	0.47-1.10	≤0.372	11 (78.6%)	3 (21.4%)	0.0066*
				>0.372	93 (98.9%)	1 (1.1%)	

These findings suggest that the risk of peripheral neuropathy increases when the AST count is below 22.0 IU/L.

Hand-foot syndrome

Monocyte count (10^2^/μL) at the beginning of one cycle was significantly higher in the Grade ≥ 1 group (7.60 ± 8.50) than the Grade 0 group (4.23 ± 1.88) (p = 0.0477) (Table 2). Red blood cell count (10^4^/μL) at the start of one cycle was significantly lower in the Grade ≥ 1 group (353.91 ± 107.37) than in the Grade 0 group (413.28 ± 57.19) (p = 0.0472) (Table 2). Alb levels at the start of one cycle were significantly lower in the Grade ≥ 1 group (3.85 ± 0.23 g/dL) than in the Grade 0 group (4.07 ± 0.37 g/dL) (p = 0.0219) (Table 2). Furthermore, AST (19.33 ± 5.19 IU/L) and ALT (15.08 ± 9.41 IU/L) levels were lower in the Grade ≥ 1 group than the Grade 0 group (30.66 ± 53.64 IU/L and 23.29 ± 14.30 IU/L, respectively) (p = 0.0469 and p = 0.0090) (Table 2). Multivariable logistic regression analysis showed that red blood cells, monocytes, Alb, AST, and ALT were not significantly associated with the development of hand-foot syndrome as independent factors (p > 0.05) (Table 3).

No predictive factors associated with an increased risk of hand-foot syndrome were identified.

Nausea

Among the predictors of nausea, a significant difference in alcohol consumption history (p = 0.0240) was observed in patient information at the start of the first cycle (Table 1). Basophils (10²/μL) at the beginning of one cycle were significantly higher in the Grade ≥ 1 group (0.56 ± 0.49, n = 9) than in the Grade 0 group (0.28 ± 0.22, n = 99) (mean difference: 0.28, 95% CI: 0.04-0.60, p = 0.0297) (Table 2). NLR at the beginning of one cycle was significantly higher in the Grade 0 group (2.31 ± 1.10) than the Grade ≥ 1 group (2.80 ± 0.53) (p = 0.0310) (Table 2). Multivariate logistic regression analysis identified occasional drinking (OR = 10.3125, 95% CI: 1.5016-206.3543, p = 0.0157) and basophils (OR = 12.0921, 95% CI: 1.7492-114.2699, P = 0.0138) as independent factors associated with the development of nausea (Table 3). On the other hand, no significant association was found with drinking several times a week, daily drinking, and NLR (Table 3).

The predictive performance of basophils was evaluated using ROC curve analysis, yielding an AUC of 0.71998, indicating acceptable predictive ability (Table 4). The cut-off value for basophils determined by the ROC curve was 0.28×10^2^/μL (95% CI: 0.3-0.7) (Table 4). Thus, a basophil count ≥ 0.28×10^2^/μL was significantly associated with Grade ≥ 1 nausea (p = 0.0137).

The risk of nausea increases when the basophil count is above 0.28×10^2^/μL.

Anorexia

Regarding predictors of anorexia, significantly higher basophils (10^2^/μL) were found at the start of one cycle in the Grade ≥ 1 group (0.52 ± 0.40) than the Grade 0 group (0.28 ± 0.22) (p = 0.0018) (Table 2). PLR at the beginning of one cycle was significantly lower in the Grade ≥ 1 group (127.98 ± 54.31) than in the Grade 0 group (178.01 ± 73.45) (p = 0.0242) (Table 2). Multivariable logistic regression analysis identified basophils (OR = 7.3613, 95% CI: 1.0601-73.0202, P = 0.0434) and PLR (OR = 0.3282, 95% CI: 0.0827-0.9694, P = 0.0430) as independent factors associated with the development of anorexia (Table 3).

The predictive performance of the model was evaluated using ROC curve analysis, yielding AUCs of 0.778 for basophils and 0.701 for PLR, indicating acceptable predictive ability (Table 4). The cut-off value for basophils determined by the ROC curve was 0.29×10²/μL (95% CI: 0.3-0.7) (Table 4). Accordingly, a basophil count ≥ 0.29×10²/μL was significantly associated with the development of Grade ≥ 1 anorexia (p = 0.0004). The cut-off value for PLR determined by the ROC curve was 152.69 (95% CI: 1.13-2.11) (Table 4). Thus, PLR ≤ 152.69 was significantly associated with Grade ≥ 1 anorexia (p = 0.0005).

These results suggest that the risk of anorexia increases when the basophil count is above 0.29×102/μL and the PLR count is below 152.69.

Diarrhea

SIRI at the start of one cycle was significantly lower in the Grade ≥ 1 group (0.44 ± 0.21) than in the Grade 0 group (0.94 ± 0.80) (p =0.0412) (Table 2). Because only one predictor was extracted, multivariable logistic regression analysis was performed using AST (p = 0.0770), which had the second smallest p value after SIRI. Multivariable logistic regression analysis identified SIRI (OR = 0.9941, 95% CI: 0.9848-0.9999, p = 0.0420) as an independent factor associated with the development of diarrhea (Table 3). On the other hand, no significant association was found with AST (p > 0.05) (Table 3).

The predictive performance of the model for SIRI was evaluated using the ROC curve, yielding an AUC of 0.80288, which indicated good predictive ability (Table 4). The cut-off value calculated from the ROC curve was 0.372 (95% CI: 0.47-1.10) (Table 4). Thus, SIRI ≤ 0.372 significantly caused Grade ≥ 1 diarrhea (p = 0.0066).

These findings suggest that the risk of diarrhea increases when the SIRI count is below 0.372.

## Discussion

This study aimed to identify adverse drug reactions and their predictors in patients with colorectal cancer treated with CapeOX. We identified patient characteristics, blood laboratory values, and inflammatory markers that serve as predictors of several adverse effects prior to treatment.

Low AST levels at the start of treatment were identified as a predictor of peripheral neuropathy. As AST is a vitamin B6-dependent enzyme, lower AST activity might reflect reduced vitamin B6-related enzymatic activity; however, this relationship remains speculative, as no clinical signs of vitamin B6 deficiency were observed before treatment initiation. Vitamin B6 is involved in amino acid metabolism and neurotransmitter synthesis (GABA, serotonin, and dopamine) and deficiency impairs neurotransmission and neuronal maintenance [3], which can result in peripheral neuropathy. Studies suggest that vitamin B6 is effective in preventing or reducing oxaliplatin-induced peripheral neuropathy. It has been reported that patients with higher blood levels of pyridoxal phosphate, the active form of vitamin B6, have a lower incidence and severity of peripheral neuropathy. In a previous study, a doubling of pyridoxal phosphate levels was associated with a 25% lower risk of developing peripheral neuropathy and a significantly lower severity score [4]. Furthermore, vitamin B6 supplementation reduces the neurotoxicity of oxaliplatin without compromising its antitumor effects [5]. Thus, a reduction in vitamin B6 may predispose patients to oxaliplatin-induced peripheral neuropathy. While elevated AST levels are generally considered clinically relevant, a large-scale cohort study of over 11,000 adults aged 70 years and older reported that low AST levels are independently associated with increased all-cause mortality and that individuals in the lowest AST quintile (8-18 IU/L for men) had a significantly higher risk of death than those in the middle quintile [6]. These findings suggest that low AST levels serve as a prognostic indicator of poor outcomes and that not only elevated but also reduced AST values may be clinically significant, particularly in older populations. AST may reflect vitamin B6 status in the patient's nervous tissue and be indirectly related to the pathogenesis of peripheral neuropathy.

When using “non-drinkers” as a reference group, being an occasional drinker was identified as a significant predictor of chemotherapy-induced nausea, whereas no significant association was found with drinking several times per week or daily. A previous meta-analysis reported that individuals with a history of alcohol consumption had a lower risk of chemotherapy-induced nausea than non-drinkers [7]. Furthermore, a review reported that patients with low alcohol intake had a higher risk of nausea, suggesting that a consistent drinking pattern may confer a protective effect [8]. To date, no studies have specifically examined the relationship between the frequency of alcohol consumption and nausea in patients receiving CapeOX therapy. This remains a topic for future investigation.

A high basophil count was a common predictor of nausea and anorexia. There are no previous studies that reported a direct relationship between high basophil levels before treatment and nausea and anorexia after chemotherapy. Basophils are a type of white blood cell involved in allergic and inflammatory reactions that release chemical transmitters such as histamine and leukotrienes. These substances may affect gastrointestinal motility and secretion and may be associated with symptoms of nausea and anorexia [9]. Further studies are needed to investigate the association between high basophil levels and nausea and anorexia.

In the present study, low PLR was associated with anorexia. There are no previous studies reporting that low pretreatment PLR is directly associated with anorexia after chemotherapy for patients with colorectal cancer. PLR is a blood biomarker that has attracted attention as an indicator of inflammatory status and immune function [10]. In general, a high PLR value suggests hyperinflammation or malignant tumor progression; thus, lymphocyte count may be depleted due to an active inflammatory response or markedly decreased due to infection or malignant tumor progression [11]. In particular, lymphocytopenia is often observed in immunosuppressed states and advanced cancer patients, and PLR is often high [12]. In the present study, we found significant differences in PLR but no significant differences in platelet and lymphocyte counts that constitute PLR. This suggests that small changes in platelet and lymphocyte counts are manifested as changes in PLR, which may sensitively reflect subtle changes in immune status and inflammatory balance [13]. Studies examining the clinicopathologic and prognostic significance of PLR in gastric cancer patients have also reported that a reduced pretreatment PLR is associated with higher responsiveness to chemotherapy in patients with advanced gastric cancer [14,15]. Peng et al. reported that patients with advanced gastric cancer and low PLR were more responsive to treatment; however, low PLR was also associated with a deterioration in general condition, including worsening performance status and decreased appetite [15]. In particular, the incidence of decreased appetite was significantly higher in the low PLR group (48.7%), suggesting that PLR may be useful as an indicator of systemic status. Wu et al. investigated the relationship between preoperative PLR and NLR and chemotherapy sensitivity and prognosis in patients with colorectal cancer and reported that the response rate to chemotherapy was significantly higher, especially in the low PLR group, and was also associated with longer progression-free survival and overall survival [16]. The high responsiveness to chemotherapy may be indirectly related to anorexia; however, further validation is needed.

Low SIRI was identified as a predictor of diarrhea; however, no reports have examined the relationship between SIRI and the development of diarrhea associated with CapeOX therapy. SIRI is calculated from peripheral blood neutrophil, monocyte, and lymphocyte counts and serves as an indicator of systemic inflammation and immune balance. Its prognostic significance has been demonstrated in multiple types of cancer. A study examining the prognostic value of SIRI in patients with colorectal cancer found that overall survival and progression-free survival were significantly shorter in a high SIRI group; moreover, SIRI was more prognostically accurate than other inflammatory markers [17]. In colorectal cancer, high SIRI has been reported to be associated with shorter overall survival and disease-free survival [18]. A study examining the association between SIRI and tumor response after chemotherapy as a prognostic factor in metastatic pancreatic cancer found that patients with pre-treatment SIRI ≥ 2.3 had shorter overall survival [19]. SIRI is a useful prognostic marker in metastatic pancreatic cancer and elevated SIRI after treatment is associated with disease progression and poor prognosis [20]. Thus, patients with low pre-treatment SIRI may have a better prognosis and a higher response to treatment. A stronger response to chemotherapy may also enhance mucosal toxicity, leading to a higher incidence of diarrhea. Therefore, the development of diarrhea in patients with low SIRI may reflect increased chemosensitivity rather than poor prognosis. SIRI evaluation of patients receiving CapeOX therapy may be useful for stratifying the risk of diarrhea adverse effects and personalized medicine. Further studies are needed.

These results provide important insights for personalized medicine in patients undergoing CapeOX therapy. Assessment of patient characteristics and blood laboratory values prior to treatment may allow prior identification of patients at high risk for certain adverse effects and the development of appropriate preventive measures and management strategies.

Although some of our findings differ from those of previous studies, several factors may explain these discrepancies. Variations in patient background, such as age, nutritional and inflammatory status, and comorbidities, may have influenced the association between baseline laboratory parameters and adverse effects. Differences in treatment intensity, dose modification criteria, and evaluation timing across institutions may also contribute to inconsistent results. In addition, as this was a single-center, exploratory study with a limited sample size, random variation and unmeasured confounders cannot be completely ruled out. Therefore, these discrepancies are likely attributable to both biological and methodological differences rather than being mere study limitations. Future multicenter prospective studies with standardized treatment protocols and longitudinal assessments are warranted to validate these findings and clarify the underlying mechanisms.

This study has several limitations. First, as this was a single-center, retrospective study; therefore, the generalizability of the findings should be interpreted with caution. Second, the study was limited to assessing adverse effects at the end of one cycle, so the long-term adverse effect profile could not be examined. Further prospective studies are needed to verify the validity of these predictors and intervention studies are needed to assess prevention and management of adverse effects. More detailed exploration of predictive factors, such as molecular biological markers and genetic polymorphisms, may also be necessary. Third, as a statistical limitation, the present study was an exploratory analysis, in which many variables were first assessed by univariate analysis before proceeding to multivariate analysis, potentially increasing the risk of alpha error due to multiple comparisons. Fourth, the small number of cases for certain adverse drug reactions, including diarrhea and nausea, raises concerns regarding the stability and reliability of the logistic regression estimates. Fifth, the AUCs obtained by ROC analysis were moderate, which limits the accuracy of the prediction model. Nevertheless, this study presents a hypothesis that may be clinically useful as an exploratory analysis and is a valuable starting point for future validation studies.

The results of this study may contribute to improved management of adverse effects in colorectal cancer patients receiving CapeOX therapy, leading to improved patient quality of life and optimized treatment.

## Conclusions

Some adverse effects of CapeOX therapy may be predicted using routine pre-treatment laboratory data, including inflammatory and nutritional markers. The identification of such predictors could allow clinicians to stratify patients according to their risk of developing specific adverse effects and to implement individualized preventive or supportive care strategies before initiating treatment. These findings highlight the potential utility of simple, routinely available biomarkers in optimizing patient safety and maintaining treatment adherence. Although this study was exploratory and limited by its single-center, retrospective design, it provides valuable hypotheses for future prospective validation studies. Further research focusing on molecular or genetic predictors, as well as interventional approaches to mitigate adverse effects, will be essential to advance personalized medicine in colorectal cancer chemotherapy.
